# Fractionation Mapping of the Ganglionated Plexi for Cardioneuroablation

**DOI:** 10.19102/icrm.2021.120405

**Published:** 2021-04-15

**Authors:** Leah A. John, Andin Mullis, Joshua Payne, Roderick Tung, Tolga Aksu, Jeffrey R. Winterfield

**Affiliations:** ^1^Division of Cardiology, Medical University of South Carolina, Charleston, SC, USA; ^2^The University of Chicago Medicine Center for Arrhythmia Care, Pritzker School of Medicine, Chicago, IL, USA; ^3^Department of Cardiology, Kocaeli Derince Training and Research Hospital, University of Health Sciences, Kocaeli, Turkey

**Keywords:** Cardioinhibitory, cardioneuroablation, ganglionated plexi, high-density mapping, vasovagal syncope

## Abstract

Cardioneuroablation (CNA) is an emerging technique used to treat patients with vasovagal syncope (VVS). We herein describe a case of CNA targeting the atrial ganglionated plexi (GPs) based upon anatomical landmarks and fractionated electrogram (EGM) localization in a 20-year-old healthy female who presented to our center with malignant VVS and symptomatic sinus pauses, the longest of which measured 10 seconds. She underwent acutely successful CNA with a demonstration of vagal response noted following ablation of the left-sided GPs, and tachycardia was noted with right-sided GP ablation. All GP sites were defined by anatomical landmarks and EGM analysis. By using the fractionation mapping software of the EnSite Precision™ cardiac mapping system (Abbott, Chicago, IL, USA) with high-density mapping, fragmented EGMs were successfully detected in each GP site. One month after vagal denervation, no recurrent syncopal episodes or sinus pauses had been recorded. Longer-term follow-up with an implantable loop recorder is planned. Broadly, we performed CNA in a patient with VVS by combining high-density mapping and fractionation mapping software in a novel approach, which allowed us to detect fractionation in all GP sites and demonstrate an acute vagal response. This workflow may facilitate the introduction of a standardized technique suitable for widespread use.

## Introduction

Vasovagal syncope is the most common cause of transient loss of consciousness.^1^ Refractory syncopal episodes can cause serious syncope-related injury and can be debilitating for patients with such episodes.^2^ Cardioneuroablation (CNA) is an emerging and promising technique to treat patients with both types of VVS.^2–6^ We describe a case of CNA targeting the atrial ganglionated plexi (GPs) based on anatomicallandmarks and fractionated electrogram (EGM) characteristicsin a young patient with cardioinhibitory syncope.

## Case presentation

### Patient characteristics

A 20-year-old healthy female presented to our center with malignant VVS and symptomatic sinus pauses. An implantable loop recorder (ILR) showed a total of 444 pauses over 18 months, with the longest recorded pause measuring 10 seconds in length and occurring approximately one month prior to the index presentation at our center. She had experienced two prior episodes of syncope preceded by a very brief prodrome, and her index episode had resulted ina motor vehicle accident.A trial of fludrocortisone failed to improve symptoms and resulted in side effects. Head-up tilt-table testing failed to elicit symptoms. She was referred to our group for cardiac pacing, but, following extensive research into therapies available for VVS, the patient elected to proceed with CNA with complete understanding of the novel and experimental nature of this treatment.

### Procedural characteristics

The patient was brought to the electrophysiology (EP) laboratory and sedated with a combination of propofol, fentanyl, and ketamine administered by an anesthesia provider. The baseline rhythm was normal sinus, with a heart rate of 80 to 90 bpm after anesthesia. The baseline cycle length was 670 ms, and a preprocedural EP study showed normal sinoatrial nodal function and normal atrioventricular nodal conduction times with an A–H interval of 63 ms and an H–V interval of 38 ms, respectively. The activated clotting time was maintainedat greater than 350 seconds with intravenous heparin prior to transseptal catheterization.

**Figure 1:**
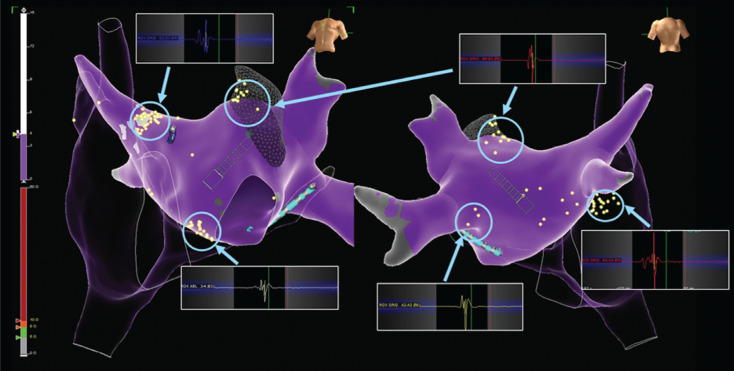
Fractionation maps of the left and right GPs with associated fractionation signals at GP sites.

A multielectrode grid catheter (Advisor™ HD Grid Mapping Catheter, Sensor Enabled™; Abbott, Chicago, IL, USA) was used to create a map of both the right and left atria to collect points using a three-dimensional electroanatomical mapping system (EnSite Precision™ system, research version; Abbott). Bipolar recordings were filtered at 200 to 500 Hz.^4,7^ Specifically, the incorporated fractionation mapping tool was used to identify regions with electrograms with multiple deflections, at a setting of 3, to identify potential GP sites in known anatomic regions^6^ **(Figure 1)**. All GP targets in both atria were identified using fractionation scoring and anatomical landmarks. Radiofrequency (RF) ablation was applied to the left-sided GPs, beginning with the left superior GP (LSGP), followed by the ridge of Marshall GP and then the left inferior GP (LIGP). Vagal responses were observed during ablation in the region of the LSGP and the LIGP at 30 to 35 W with one-minute applications (Tacticath™ SE; Abbott). Following ablation of the left-sided GP regions, the resting heart rate increased from 90 to 105 bpm. We then targeted the right superior GP (RSGP), followed by the right inferior GP (RIGP) and Q1 and then the superior vena cavic–aortic GP via the right atrium, where tachycardia was observed during RF applications **(Table 1)**. We continued with ablation at the superior vena cava–aorta junction. At the completion of RF ablation, 2 mg of atropine was administered intravenously without an effect on heart rate, which served as the procedural endpoint. The total ablation and procedure times were 11.4 and 146 minutes, respectively.

### Follow-up

**Figure 2:**
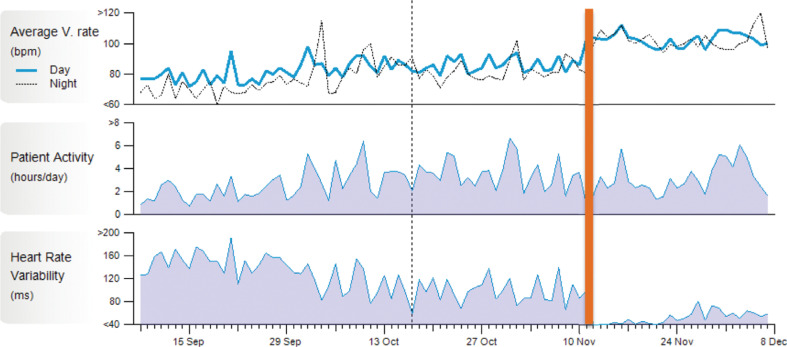
Recordings from the patient’s ILR. A significant increase in the average resting heart rate and equalization of day and night heart rates occurred after ablation. Heart rate variability was also significantly reduced after ablation. The orange line denotes the occurrence of the CNA procedure on November 11, 2020; preablation and postablation recordings are visible on either side of this line.

One month after atrial vagal denervation, there were no recurrent syncopal episodes or recurrent sinus pauses. Recordings from the ILR revealed an increased average resting heart rate and significantly reduced heart rate variability after ablation **(Figure 2)**. Longer-term follow-up with an ILR is planned with remote telemetry and routine clinical follow-up.

## Discussion

VVS is traditionally managed by medications, device placement, and/or behavioral therapy.^1^ Catheter-based CNA is emerging as a novel treatment for both cardioinhibitory and vasodepressor VVS.^2–6^ Pachon et al. first reported successful vagal denervation via RF catheter ablation targeting GPs in a cohort of 21 patients, including six with neurally mediated reflex syncope, seven with functional high-grade atrioventricular block, and 13 with sinus node dysfunction. Success was demonstrated in all cases with relief of symptoms at a mean follow-up point of 9.2 months.^5^

To the best of our knowledge, this is the first case to use the fractionation mapping software of the EnSite Precision™ mapping system with high-density mapping to successfully detect fragmented EGMs in each GP **(Figure 1)**. The identification of these fractionated potentials, also known as atrial fibrillation nests, was described as early as the 1990s, and ablation at these sites resulted in significant vagal denervation.^8,9^ This work ultimately led to the first published study by Pachon et al. describing CNA as a novel treatment targeting atrial fibrillation nests in the management of patients with adverse parasympathetic autonomic influence.^5^

The visual analysis and interpretation of EGMs is influenced by interoperator variability, which can be problematic at the beginning of the learning curve and prevent generalizability of this approach in GP localization. A unique aspect of our case was the use of the EnSite Precision™ system’s fraction mapping tool. The use of a standardized software program may allow this limitation to be overcome and for the standardization of this technique, regardless of operator experience in EGM analysis. Previous use of this software was attempted by Aksu et al.,^6^ who described successful vagal denervation using fraction mapping software to localize GPs during sinus rhythm. However, they used an ablation catheter for mapping, and fragmented areas were missed in some locations. The use of high-density mapping in combination with a uniform mapping software program may facilitate the introduction of a new workflow for electroanatomically guided CNA.

Although we demonstrated acutely successful vagal denervation with a positive vagal response with the left-sided GP and an elevated baseline heart rate at the end of the case, we did not observe a vagal response with the right-sided GP, instead recording an increase in the sinus rate during ablation of the RSGP, which remains consistent with the findings described by Aksu et al.^7^ Contrary to previous reports, we detected a heart rate increase relative to the baseline rate following the ablation of left-sided GPs.^2^ This difference in response between the right- and left-sided GPs may be attributable to varying ratios of parasympathetic, sympathetic, and sensory neurons in each of the GPs and individual differences among them.^7^

An important limitation of CNA to consider in this case is the lack of direct control of vagal denervation. The atropine test performed at the end of this procedure failed to alter the heart rate, suggestive of a denervation effect, but a hard endpoint defined by direct control of vagal output remains elusive. However, the short-term result **(Figure 2)** suggests a therapeutic vagal denervation effect had occurred.

## Conclusions

CNA may be a promising approach for a cohort of patients with refractory VVS. Still, standardization of procedural endpoints and the ablation approach is necessary to optimize the efficacy and uniformity of CNA. High-density mapping in combination with an auto-algorithm fraction mapping system may allow for use of a new, standardized technique to localize GPs as a complement to anatomically-guided approaches. Prospective, randomized, controlled, and multicenter studies are warranted to further evaluate the safety and efficacy of this therapy.

## Figures and Tables

**Table 1:** R–R Interval Changes During RF Ablation at Different GP Sites

Anatomic Location	Preablation CL	Postablation CL	R–R Interval Change*
LIGP	660 ms	711 ms	↑ 7.7%
LSGP	650 ms	715 ms	↑ 10%
RSGP	570 ms	520 ms	↓ 8.7%
RIGP	580 ms	570 ms	↓ 1.7%
SVC-Ao GP	587 ms	565 ms	↓ 3.7%

CL: cycle length; LIGP: left inferior ganglionated plexus; LSGP: left superior ganglionated plexus; RIGP: right inferior ganglionated plexus; RSGP: right superior ganglionated plexus; SVC-Ao: superior vena cava–aorta.

*RF ablation at the LIGP and LSGP led to a vagal response related–increase in the cycle length, while ablation at the RSGP, RIGP, and SVC-Ao GP led to a decrease in the cycle length.
